# KBG syndrome involving a single-nucleotide duplication in *ANKRD11*

**DOI:** 10.1101/mcs.a001131

**Published:** 2016-11

**Authors:** Robert Kleyner, Janet Malcolmson, David Tegay, Kenneth Ward, Annette Maughan, Glenn Maughan, Lesa Nelson, Kai Wang, Reid Robison, Gholson J. Lyon

**Affiliations:** 1 Stanley Institute for Cognitive Genomics, Cold Spring Harbor Laboratory, Cold Spring Harbor, New York 11724, USA;; 2 Genetic Counseling Graduate Program, Long Island University (LIU), Brookville, New York 11548, USA;; 3 Affiliated Genetics, Inc., Salt Lake City, Utah 84109, USA;; 4 Epilepsy Association of Utah, West Jordan, Utah 84088, USA;; 5 KBG Syndrome Foundation, West Jordan, Utah 84088, USA;; 6 Zilkha Neurogenetic Institute, University of Southern California, Los Angeles, California 90089, USA;; 7 Department of Psychiatry & Behavioral Sciences, Keck School of Medicine, University of Southern California, Los Angeles, California 90033, USA;; 8 Utah Foundation for Biomedical Research, Salt Lake City, Utah 84107, USA

**Keywords:** absent speech, autism, bilateral single transverse palmar creases, broad nasal tip, clinodactyly of the 5th finger, developmental regression, generalized tonic–clonic seizures on awakening, intellectual disability, moderate, low CSF 5-methyltetrahydrofolate, pes planus, short philtrum, short toe

## Abstract

KBG syndrome is a rare autosomal dominant genetic condition characterized by neurological involvement and distinct facial, hand, and skeletal features. More than 70 cases have been reported; however, it is likely that KBG syndrome is underdiagnosed because of lack of comprehensive characterization of the heterogeneous phenotypic features. We describe the clinical manifestations in a male currently 13 years of age, who exhibited symptoms including epilepsy, severe developmental delay, distinct facial features, and hand anomalies, without a positive genetic diagnosis. Subsequent exome sequencing identified a novel de novo heterozygous single base pair duplication (c.6015dupA) in *ANKRD11,* which was validated by Sanger sequencing. This single-nucleotide duplication is predicted to lead to a premature stop codon and loss of function in *ANKRD11,* thereby implicating it as contributing to the proband's symptoms and yielding a molecular diagnosis of KBG syndrome. Before molecular diagnosis, this syndrome was not recognized in the proband, as several key features of the disorder were mild and were not recognized by clinicians, further supporting the concept of variable expressivity in many disorders. Although a diagnosis of cerebral folate deficiency has also been given, its significance for the proband's condition remains uncertain.

## INTRODUCTION

Whole-exome sequencing (WES) is a method that mainly targets regions of the genome that code for proteins and is useful for detecting disease-contributing variants in genes associated with rare genetic syndromes (Bamshad et al. 2011; Lyon and Wang 2012; Lyon and Segal 2013; Biesecker and Green 2014; Chong et al. 2015; Guo et al. 2015; He et al. 2015; Lyon and O'Rawe 2015). Here we report our efforts in phenotypic characterization and molecular diagnosis of a previously undiagnosed pediatric patient. We report the identification of a de novo mutation in *ANKRD11*, which led to the recognition of KBG syndrome (Ockeloen et al. 2015; Walz et al. 2015) in the sequenced proband. KBG syndrome (OMIM #148050) is a rare, but increasingly recognized, autosomal dominant genetic condition. It was first described in 1975 and is characterized by craniofacial features, hand abnormalities, macrodontia, and neurological involvement including developmental delay and epilepsy (Herrmann et al. 1975). The syndrome's name was derived from the last names of the first three families found to have this syndrome (Herrmann et al. 1975). Our primary goal in reporting this detailed case description is to provide rich phenotypic information that is still typically missing from many studies (Hall 2003), thus enabling a better description and accounting of the tremendous variable expressivity (Lyon and O'Rawe 2015) and stochastic developmental variation (SDV) (Vogt 2015) in all diseases.

## RESULTS

### Clinical Presentation and Family History

**Figure 1.**
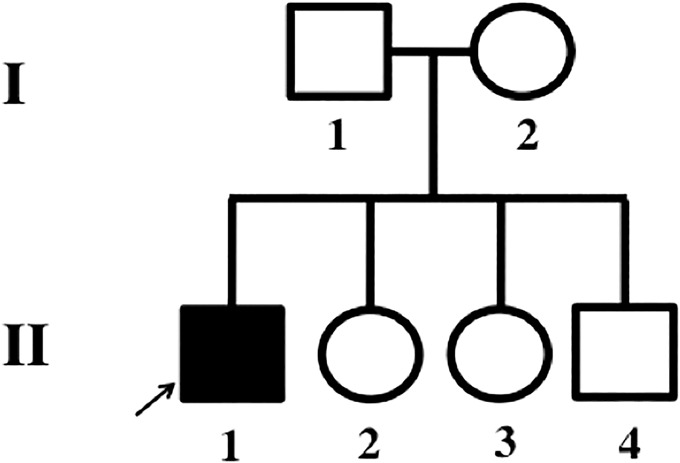
Pedigree: II-1, the affected proband (age 13), is the son of an unaffected, nonconsanguineous couple. The proband has two younger unaffected sisters (10-yr-old and 4-yr-old) and one younger unaffected brother (2-yr-old).

The proband was born to a nonconsanguineous couple, who had an unremarkable pregnancy history; however, at birth a large fontanel was reported. Parents and siblings were healthy, and no significant family history was reported (Fig. 1). The proband had his first epileptic episode at 3 years of age. After this episode, he lost all speech, began exhibiting autistic behavior, and also started to have frequent generalized tonic–clonic seizures. Over time, tonic, atonic, mild clonic, complex partial, myoclonic, and gelastic seizures were reported in the proband. Other developmental skills, including throwing a ball, responding to his name, feeding himself with utensils, and self-care skills were lost by 4 years of age. No significant conductive hearing loss, heart abnormalities, or delayed bone age were found in the proband at that age. The Social Communication Questionnaire (SCQ) (Corsello et al. 2007) filled in by the parents was scored at 23, indicating the need for additional comprehensive evaluation by a specialist such as a child psychiatrist, psychologist, or developmental pediatrician trained in diagnosing autism spectrum disorder (ASD). He attended a week in an autism evaluation classroom where he was diagnosed with ASD and considered severe and qualified for every service offered. However, the only services that he has received were those delivered in his school, partly because of complications from the severity of his seizures and also because of the lack of availability of such services.

**Figure 2.**
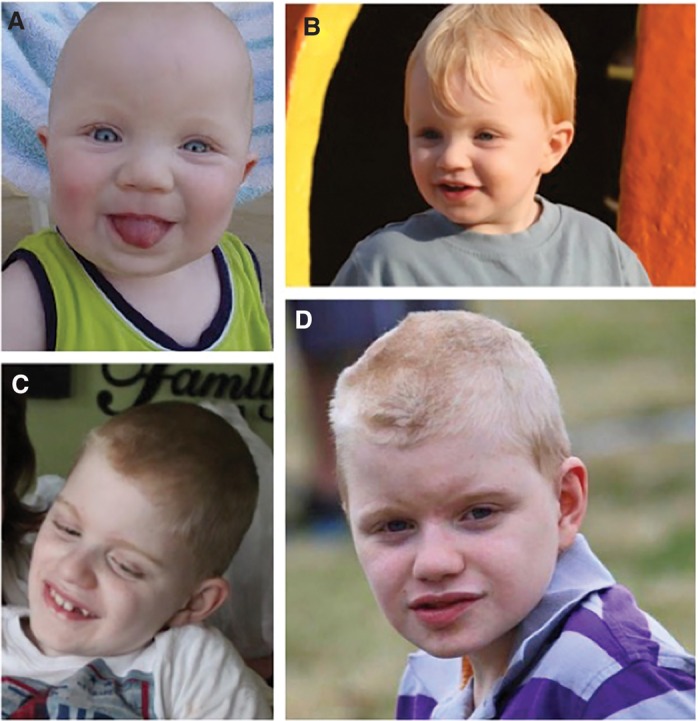
Pictures of phenotype of proband throughout childhood, at (*A*) 6 months old, (*B*) 4 years old, (*C*) 9 years old, and (*D*) 13 years old. Facial characteristics include rounded face, bushy eyebrows, broad nasal tip, short philtrum, thick lips, and prognathism.

**Figure 3.**
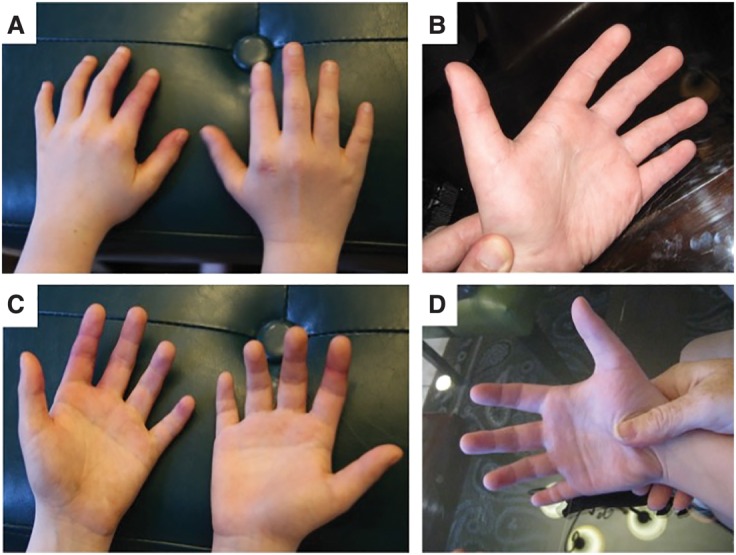
Hand anomalies include bilateral clinodactyly of the fifth finger (*A*–*D*), brachydactyly (*A*–*D*), and bilateral single transverse palmar creases (*B*–*D*).

The proband was evaluated (by G.J.L.) at 11 years of age. He has several neurological and craniofacial abnormalities including epilepsy, ventriculomegaly, relative macrocephaly, prominent forehead, low hairline, thick eyebrows, wide-set eyes, and full lips (Fig. 2; Supplemental File 1). Hand and foot abnormalities included clinodactyly of the fifth digit, bilateral single transverse palmar creases, brachydactyly (Fig. 3), and flat feet. He was primarily nonverbal during the course of the evaluation and also exhibited decreased eye contact and social engagement, relative to his siblings. A summary of his clinical features is shown in Table 1.

**Table 1.** Summary of the clinical features found in this proband

Features (Human Phenotype Ontology Nos)	Proband
Facial dysmorphism	
Large fontanelle (HP:0000239)	+
Rounded face (HP:0000311)	+
Bushy eyebrows (HP:0000574)	+
Broad nasal Tip (HP:0000455)	+
Short philtrum (HP:0000322)	+
Full/thick lips (HP:0012471)	+
Cupid bow upper lip (HP:0002263)	+
Macrodontia of upper central incisors (HP:0000675)	+
Prognathism (HP:0000303)	+
Developmental/intellectual disability	
Intellectual disability (HP:0001249)	+
Developmental regression	+
Developmental delay prior to regression	+
Absent speech (HP:0001344)	+
Skeletal	
Clinodactyly of the fifth finger (HP:0004209)	+
Brachydactyly (HP:0009803)	+
Bilateral single transverse palmar creases (HP:0007598)	+
Short toes (HP:0001831)	+
Pes planus (HP:0001763)	+
Neurological	
Seizures (T/C, atonic, complex, partial, tonic, gelastic) (HP:0001250)	+
Growth	
Currently short stature (10th percentile) (HP:0004322)	+
Behavioral	
Autistic behavior (HP:0000729)	+
Congenital birth defects	
Congenital heart defect	−
Surgeries: ear tubes, broken jaw	+
Cryptorchidism	−
Palatal defects	−
Miscellaneous	
Low CSF 5-methyltetrahydrofolate (HP:0012446)	+
Hearing loss	−

CSF, cerebrospinal fluid.

In 2011, during the course of his clinical workup, the proband was found to have a somewhat low level of 5-methyltetrahydrofolate (5-MTHF) in his cerebrospinal fluid (CSF) (32 nmol/L, where the reference range is 40–128 nmol/L). The concentration of pyridoxal 5′-phosphate in the CSF was within the reference range, 25 nmol/L, with range 10–37 nmol/L. With these results, a diagnosis of cerebral folate deficiency was given, and it was recommended that he begin treatment with oral folinic acid. The proband has been receiving folinic acid (leucovorin, 50 mg/day) since that time, and a repeat CSF analysis in 2013 showed an increased level of 5-MTHF (75 nmol/L, where the reference range is 40–128 nmol/L). He was even treated clinically with a 5-d course of intravenous immunoglobulin (IVIG), despite the fact that there is very little in the medical literature demonstrating the efficacy of this. He was later shown to have 1.52 pmoles/mL serum of folate receptor (FR) blocking antibody, where the range of these values is as follows: <0.5, low; >0.5–1.0, medium; >1, high. He was shown to have an FR binding autoantibody titer of 2.37 pmoles IgG/mL in serum, where the range for FR binding autoantibody titers is <1, low; >1–5, medium; >5, high. Binding autoantibodies against the FR recognize epitopes other than the folate binding site on the FR (Ramaekers et al. 2007, 2016). It should be noted that recent work has demonstrated that there is a lack of control data for 5-MTHF in healthy children, and the limited number of longitudinal measurements has shown considerable variability even in healthy children (Shoffner et al. 2016). Future studies should carefully measure in a longitudinal fashion in many more healthy children not only 5-MTHF in CSF but also FR blocking and binding autoantibodies, particularly given that the prevalence of these antibodies has not been ascertained or published in thousands of healthy children.

Some patients with cerebral folate deficiency (CFD) have been reported to have mutations in the folate receptor 1 (*FOLR1*) gene (MIM *136430) with extremely low levels of 5-MTHF in CSF (Cario et al. 2009; Delmelle et al. 2016), but we did not find any mutation in this gene in this proband. CFD is reported to also be caused by the interruption of folate transport across the blood–brain barrier due to folate receptor autoantibodies (FRAs) (Frye et al. 2013). CFD has been reported to be associated with neurological findings including seizures, spastic paraplegia, cerebellar ataxia, dyskinesia, and developmental regression, and recent cases have described ASD (Frye et al. 2013), although much of this is nonspecific and not correlated in any way with the severity of any of the symptoms (Mangold et al. 2011).

The proband has also been treated with various antieplileptic drugs (AEDs) including lamotrigine (Lamictal, 400 mg/day), which has been the most effective antiepileptic drug to control his seizures as per family report, and recently 1.5 mL twice daily of 100 mg/mL Epidiolex (cannabidiol) has also been reported by the family to reduce his frequency of seizures (Devinsky et al. 2015; Filloux 2015). A caveat is that these treatments were not administered in a blinded fashion nor was there any period of treatment with a placebo. In addition, the strength of evidence from clinical trials for efficacy of these medications for epilepsy is strongest for lamotrigine (Kwan et al. 2011; Li et al. 2012; Liu et al. 2016), whereas the evidence is currently much weaker regarding the efficacy for autism disorder for folinic acid (Ramaekers et al. 2016) and/or IVIG (Wong and White 2015). As of the publication of this study, there are ongoing clinical trials for cannabidiol (Devinsky et al. 2015; Filloux 2015).

### Genomic Analyses

Blood and saliva samples from the proband as well as his parents and siblings were used as samples to be sequenced. These samples were sent to Affiliated Genetics in Salt Lake City, Utah, where genomic DNA was extracted and exons sequenced using the Life Technologies Ampliseq Exome RDY kit and the Life Technologies Proton sequencing system (see Methods). These targeted regions were sequenced using the Ion Proton sequencing system using Ion Hi-Q Chemistry with 200-bp reads. The DNA sequencing data were compared with the UCSC hg19 reference sequence using several methods of analysis (see Methods). A summary of variants called for all individuals in the family are described in Table 2, and coverage and mapping statistics are shown in Table 3. These analyses included in-house protocols and several commercial software packages including Tute Genomics, Omicia Opal, and Cartagenia v4.1, along with the use of an OTG-snpcaller pipeline (see Methods). The various analyses do provide a more comprehensive and in-depth approach to the data, although reducing the false-negative rate also can lead to an elevation of the false-positive rate (O'Rawe et al. 2013, 2015).

**Table 2.** Count of single-nucleotide polymorphisms (SNPs), insertions and deletions (indels), and the total number of variants for each sequenced family member

Individual	Number of single-nucleotide polymorphisms	Number of insertions/deletions	Total number of variants
Proband	21,014	769	21,783
Mother	21,224	1011	22,235
Father	20,203	953	21,156
Sister 1	21,030	959	21,989
Sister 2	21,458	1046	22,504
Brother	20,163	1253	21,416

**Table 3.** Sequencing statistics including average read depth in exonic regions, number of reads, and percent of reads mapped for each individual sequenced

Individual	Average read depth	Number of reads	Reads mapped (%)
Proband	113.71	41,047,051	98.57
Mother	107.59	38,625,292	98.97
Father	74.70	26,177,859	98.69
Sister1	95.57	33,990,105	98.91
Sister2	127.92	44,992,904	98.86
Brother	70.07	27,394,501	96.08

As one example, for the OTG-snpcaller pipeline, for each individual, the final variant call format (VCF) file contained 20,000 to 25,000 variants, of which around 300 to 400 variants were found to be autosomal recessive (i.e., heterozygous in both parents and homozygous only in the proband). More than 1000 variants were recognized as de novo, which is well above the expected number of de novo mutations found in WES (Bamshad et al. 2011; Kong et al. 2012); this is because there are a significant number of false-positives in this data set, as we have adopted lenient filters to reduce the rate of false negatives (O'Rawe et al. 2013, 2015). Although we can easily reduce the number of variants with stringent read quality and coverage cutoffs, we have used more lenient filters and then prioritized variants based on their phenotypic relevance.

**Figure 4.**
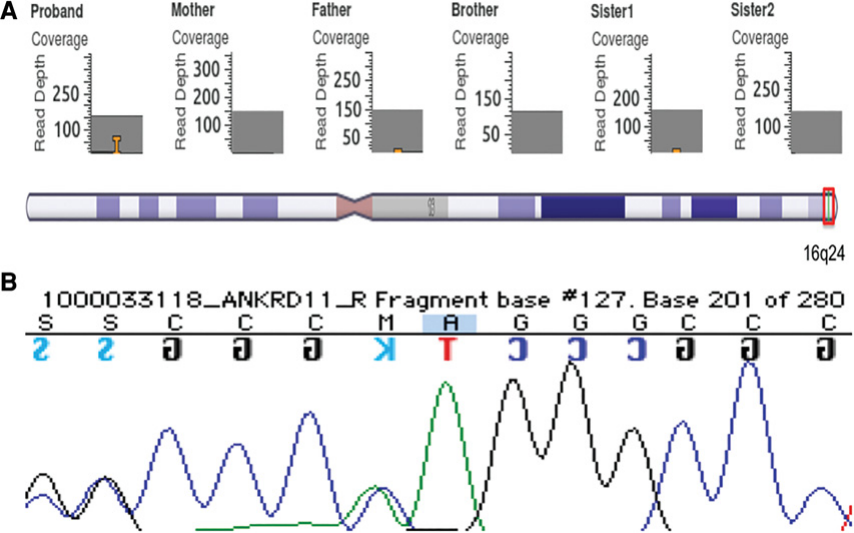
GenomeBrowse output for the proband. The orange T in the proband's nucleotide indicates a heterozygous thymine duplication in Chromosome 16, position 89,346,935. This insertion appears to be supported by more than 20 reads and is likely a true-positive mutation. (*A*) None of the other family members appears to have this mutation, indicating that it is likely de novo. The small orange bar on the father and sister indicates one read supporting an adenine insertion and a thiamine insertion, respectively, and are considered to be sequencing errors. The red box on the cytoband shows the mutation's location on Chromosome 16. (*B*) Sanger sequencing confirms the thymine duplication.

Variants with a possible autosomal recessive inheritance pattern were examined, and there was no strong evidence found to support any of them as possible contributing mutations. These variants are provided in supplementary files (as described in Methods). For the de novo mutations, a single-nucleotide duplication of adenine (A) at position 6015 in exon 10 of *ANKRD11* (c.6015dupA , p.Gly2006Argfs*26) (Table 4; Fig. 4) was identified as the most relevant mutation. All phenotypic analysis software, including Phenolyzer (Yang et al. 2015), wANNOVAR (Chang and Wang 2012), and PhenIX (Zemojtel et al. 2014), indicated that a heterozygous frameshift mutation in *ANKRD11*, or the ankyrin repeat domain 11 gene, is likely to be a contributing factor in this individual's disease. This mutation has a Combined Annotation-Dependent Depletion (CADD) score of 32, and it is considered to be “Deleterious” by SIFT with a confidence score of 0.858 and is therefore predicted to have a severe effect on protein structure. SIFT also predicted that nonsense-mediated decay would be a likely result in the case of this variant. The Exome Aggregration Consortium (ExAC) probability of loss-of-function (LOF) intolerance (pLI) score was calculated to be 1.0, indicating that this gene is very intolerant to mutations, and previous studies have indicated that mutations in this gene often lead to haploinsufficiency (see Discussion) (Lek et al. 2016b). Other mutations in this gene have been previously identified as contributing to KBG syndrome, a rare disease that affects around 60 to 70 people worldwide (Brancati et al. 2006; Sirmaci et al. 2011; Crippa et al. 2015; Walz et al. 2015). The presence of the mutation was confirmed using Sanger sequencing (Fig. 4).

**Table 4.** *ANKRD11* variant

Chr:position GRCh37(hg19)	HGVS cDNA	HGVS protein	Type of variant	Predicted effect	Genotype	Parent of origin
Chr16:89346934	c.6015dupA	p.Gly2006Argfs*26	Duplication	Frameshift	Heterozygous	De novo

## DISCUSSION

Many syndromes affecting neurological development present with heterogeneous and nondistinct phenotypes (Lyon and O'Rawe 2015) and therefore remain undiagnosed or are misdiagnosed. The combination of whole-exome sequencing combined with detailed and standardized phenotypic documentation is a powerful method to achieve improved diagnoses, particularly in the context of large-scale genomic sequencing efforts of normal population controls (Lek et al. 2016a). In this regard, we report here a unique mutation (never seen in more than 60,000 individuals) in a highly conserved and mutation-intolerant gene, *ANKRD11*. This stands in contrast to a mildly low level of 5-MTHF detected in CSF for the proband, in the context of a lack of control data for 5-MTHF measured longitudinally in thousands of healthy children (Frye et al. 2013; Shoffner et al. 2016). It is also worth noting that, to our knowledge, previous studies proposing the efficacy of folinic acid treatment fail to compare its treatment with a control (e.g., a placebo).

**Figure 5.**
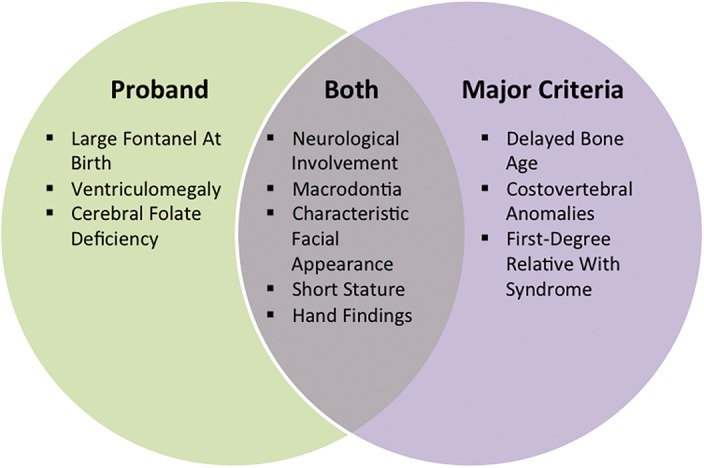
Venn diagram showing the eight major criteria suggested for KBG syndrome and the five characteristics present in the proband. For a clinical diagnosis of KBG syndrome it has been suggested that four of the eight criteria be present. We note that we have been unable to obtain precise measurements of the proband's teeth, so the mild macrodontia was noted only by visual inspection and by family report, including the observation by the parents that the proband's central incisors are larger than those of his siblings.

**Figure 6.**
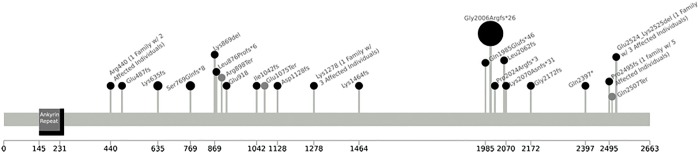
Some of the published mutations in *ANKRD11*, most of which are loss of function (LOF). Mutations represented by the black circles were those documented in individuals with KBG syndrome. The mutations represented by the gray circles are those reported in Exome Aggregration Consortium (ExAC) data. The mutation represented by the large black circle was the one found in the proband. Only the LOF mutations in ExAC were plotted. The height of each mutation varies only for the ease of showing the mutations in the figure. The image was created using the “lollipops” tool (https://github.com/pbnjay/lollipops), which retrieved domains from Pfam. Unless otherwise noted in parentheses, all mutations were found in only one individual. None of the exact same mutations have been found in more than one family. A more detailed version of this figure can be viewed by opening the file ANKRD11_Mutations_Web_Viewable.svg (Supplemental Material) in a Web browser and holding the cursor over the domain and the mutations.

Skjei et al. (2007) suggested that a clinical diagnosis of KBG syndrome can be made if the individual meets four out of the following eight major criteria: characteristic facial features, macrodontia of upper central incisors, short stature, delayed bone age, neurological involvement, hand abnormalities, costovertebral anomalies, and the presence of a family member affected with the syndrome (see Fig. 5). Facial features include hypertelorism, short nose with broad base and bulbous nasal tip, and broad bushy eyebrows (Ockeloen et al. 2015). Although the shape of the face is often described as being round, it has been noted that the shape evolves as affected children develop (Skjei et al. 2007). Hand abnormalities typically include brachydactyly, clinodactyly of the fifth digit, small hands, and nail anomalies (Skjei et al. 2007). Skeletal anomalies frequently involve the pelvis, thorax, limbs, and skull with abnormal curvature of the spine, including kyphosis and scoliosis, being reported in some cases (Skjei et al. 2007). Minor features of KBG syndrome include cutaneous syndactyly, conductive hearing loss, palatal abnormalities, cryptorchidism, webbed/short neck, strabismus, and congenital heart defects (Brancati et al. 2006). There is some phenotypic overlap with Cornelia de Lange syndrome (CdLS), although there is nothing that is necessarily pathognomonic in either disorder (Ansari et al. 2014; Parenti et al. 2015). More than 100 cases have now been reported (see Fig. 6; Ockeloen et al. 2015; Goldenberg et al. 2016); however, it is likely that KBG syndrome is underdiagnosed because dysmorphic features may be underreported, subtle, or even nonexistent, and cognitive delay can vary from mild to moderate (Crippa et al. 2015).

Individuals with KBG syndrome have been found to have heterozygous mutations leading to haploinsufficiency of the ankyrin repeat domain 11 (*ANKRD11*) gene or a 16q24 microdeletion that encompasses *ANKRD11* (Ockeloen et al. 2015). Mutations that lead to premature stop codons could trigger nonsense-mediated decay (NMD) and result in haploinsufficiency. Sporadic and familial cases of KBG syndrome have been reported, with familial cases following an autosomal dominant inheritance pattern (Zollino et al. 1994; Brancati et al. 2004; Maegawa et al. 2004; Davanzo et al. 2005; Skjei et al. 2007; Lim et al. 2014; Tunovic et al. 2014; Hafiz et al. 2015; Kim et al. 2015; Ockeloen et al. 2015). ANKRD11 is a chromatin regulator that controls histone acetylation and gene expression during neural development (Gallagher et al. 2015). There are two functional domains that act as transcriptional repressors and one domain that functions as a transcriptional promoter (Zhang et al. 2007). The majority of reported mutations in KBG syndrome result in a truncated protein that affects a domain for transcriptional repression (Crippa et al. 2015). ANKRD11 interacts with the p160 coactivator and the nuclear receptor complex, and it functions to inhibit ligand-dependent transcriptional activation by recruiting histone deacytelases (HDACs) (Sirmaci et al. 2011). Additionally, ANKRD11 was also found to play a role in enhancing the transcriptional activity of p53 (Neilsen et al. 2008). Homozygosity for a missense mutation in *ANKRD11* is embryonic lethal in mice, whereas the heterozygous mice have an osteopenia-like phenotype and craniofacial abnormalities (Barbaric et al. 2008).

This sporadic case of KBG syndrome demonstrates the importance of ongoing investigations of rare conditions. Each case reported in the literature will help to delineate the phenotypic spectrum, so that we may better identify cases in the future and determine appropriate recommendations for clinical management. Current recommendations for management of KBG syndrome include hearing tests, ophthalmologic assessments, echocardiography, an electroencephalogram (EEG), orthodontic evaluation, and skeletal investigation with special attention to spine curvatures and limb asymmetry (Brancati et al. 2006; Ockeloen et al. 2015). Additionally, this case also demonstrates the variability of the clinical manifestations of KBG syndrome, as any macrodontia in the proband was not noticeable enough to be recognized and associated with any syndrome diagnosis by any clinician, including medical geneticists, before the molecular diagnosis of the syndrome. This is consistent with the very recent suggestion that macrodontia should not be considered a “mandatory feature” of KBG syndrome (Goldenberg et al. 2016). In addition, seizures are an often reported feature of KBG syndrome, found in up to 28% of patients (Skjei et al. 2007). Typically, seizures with KBG syndrome are characterized as tonic–clonic, responsive to treatment, transient, and benign (Brancati et al. 2006; Ockeloen et al. 2015). However, in this proband, seizures have been persistent, mixed generalized, and partial, treatment-resistant, and temporally associated with developmental regression at seizure onset.

Although there is currently no cure for this disease, identifying individuals with this syndrome will not only provide a method to track the outcome of these individuals but also help provide support. For instance, the family of the affected individual created a social media group, and a KBG nonprofit foundation was specifically developed to connect families with children affected with KBG syndrome. Tracking these individuals might also identify information regarding the progression of the disease and any shared, or individual, phenotypes that might be relevant to study in the future. Of course, we look forward to the day when there is a comprehensive and interactive database that incorporates extensive phenotypic and whole-genome information while maintaining acceptable privacy standards, but no such database currently exists, although there are certainly efforts along these lines (Kibbe et al. 2015; McMurry et al. 2016).

## METHODS

### DNA Isolation and Sequencing

Genomic DNA was extracted using standard methods (Puregene, QIAGEN). The Life Technologies Ampliseq Exome RDY kit (Thermo Fisher) was used to target the exon regions. Of note, 97% of Consensus Coding Sequences with 5-bp exon padding were amplified using 294,000 primer pairs. These products were sequenced using the Life Technologies Proton sequencing system with 200-bp reads using a P1V3 chip.

### Variant Calling

The DNA sequence was aligned to the UCSC hg19 reference sequence and variants were called using the Torrent Suite software and the Torrent Variant caller. Only exonic variants and variants at the intron–exon boundary (1 or 2 nt into the intron and 1 nt into the exon) were reviewed. For each variant considered, depth of coverage was >10× and the quality score was >30. Ethnicity and variant frequency were considered during analysis. Analysis of the variants was conducted by two independent reviews using in-house protocols and two commercial software packages, Tute Genomics and Cartagenia v4.1. Pathogenic variants were confirmed by Sanger sequencing. American College of Medical Genetics and Genomics (ACMG) reporting criteria were used to evaluate variants (Richards et al. 2015).

In additional analyses, binary alignment (BAM) files from the Ion Torrent Personal Genome Machine (PGM) platform were converted to FASTQ files. Variants were called using the OTG-snpcaller pipeline, which has been reported to map a higher proportion of sequencing reads to the reference genome in comparison to other methods and result in lower error rates when analyzing sequences coming from the PGM platform (Zhu et al. 2014). Unlike other sequencing software and pipelines such as the Genome Analysis Toolkit (GATK) and FreeBayes (McKenna et al. 2010; Garrison and Marth 2012), OTG-snpcaller is specifically designed to take into account errors associated with PGM data, such as errors around homopolymers, thus increasing overall accuracy.

After the hardcoded pipeline was received from one of the authors of its paper, it was recoded (without any change to its function), so that it could be run on a computational cluster on campus. Analysis was run for each member of the proband's immediate family, including his parents and siblings. Variants were aligned to the GRCh37 assembly, as several downstream analysis tools do not yet support the new GRCh38 assembly. A VCF file containing information about each mutation was then output (Danecek et al. 2011). All programs created, or rewritten by the author (R.K.), and used for analysis were uploaded to GitHub and can be found at https://github.com/rkleyner/PGM-WES-Pipeline.

### Variant Selection and Prioritization

The resulting VCF file for each individual in the family (see Supplemental File 2) was converted into ANNOVAR files (avinput) using ANNOVAR. avinput provides information regarding chromosome number, start position, end position, reference nucleotide, alternate nucleotide, and quality scores for each variant (Wang et al. 2010). All avinput files for a particular family were then loaded into a Python program (see Supplemental File 3), which performs set intersections using DataFrame functions from the Pandas library and set functions using the Numpy library to identify de novo and autosomal recessive variants (McKinney 2010; Van Der Walt et al. 2011). Autosomal recessive variants were identified by isolating homozygous variants in the affected child, intersecting these variants with variants that were heterozygous in both parents, and subtracting variants that were homozygous in the siblings. De novo variants were identified by subtracting variants found in the parents and siblings from variants found in the proband.

The columns examined included the chromosome number, start point, end point, and zygosity of each called variant. The resulting avinput files were then output as BED files, which contain columns providing chromosome number, start point, and end point of the mutation. This process ensured that the resulting BED files contained all autosomal recessive and de novo variants that could be determined from the VCF:

$$\begin{array}{rcl} \text{autosomal recessive} & = & [(M_{\text{het}}\cap F_{\text{het}})\cap P_{\text{hom}}]-{\text{SIB}}_{\text{hom}}],\\ \text{denovo} & = & P_{\text{all}}-M_{\text{all}}-F_{\text{all}}-{\text{SIB}}_{\text{all}}, \end{array}$$

where *M* refers to the mother's variants, *F* refers to the father's variants, *P* refers to the proband's variants, and SIB refers to the sibling's variants. The subscript het refers to heterozygous variants, hom refers to homozygous variants, and all refers to all variants.

Using the GATK SelectVariants tool, these two BED files were intersected with the original VCF file two separate times, creating two VCF files, one containing only autosomal recessive variants and one containing only de novo variants.

Both these VCF files were then annotated with the Variant Effect Predictor (VEP) software (McLaren et al. 2010), which provided additional information about the variants. This annotated VCF file was then used with GEMINI (Paila et al. 2013), which is a powerful, yet flexible network that allows for organization, sorting, and filtering of variants based on VEP and additional annotations. Variants were then filtered using rarity, deleteriousness, and read quality as filter criteria.

Rarity was determined using the ExAC database (version 0.3.1), which contains population allele frequencies for exonic variants gathered from 60,706 unrelated individuals with no history of severe pediatric disease (Lek et al. 2016b). Rare variants were considered to be variants not found in ExAC. Deleteriousness was determined by CADD scores, which encompass 63 annotations to determine a variant's deleteriousness. CADD scores are based off PHRED quality scores; therefore a minimum CADD score of ≥20 or zero (as CADD was not calculated for indels), corresponding to the top 1% most deleterious variants, was selected as a cutoff (Kircher et al. 2014). Although the resulting quality scores from the OTG-snpcaller pipeline did not correspond to the standard PHRED quality score, a minimum cutoff score of ≥120 was decided after comparing variant calls with their corresponding BAM files. Chromosome number, start point, and end point columns of variants that met these three requirements were obtained using GEMINI, and the output was saved as a BED file (see Supplemental File 5). The GEMINI query used was gemini query -q “select chrom, start, end from variants where qual≥120 AND (cadd_scaled>20 OR cadd_scaled is NULL) AND in_exac=0 order by chrom, start” denovo.db

This BED file along with Human Phenotype Ontology (HPO) numbers corresponding to the proband's phenotype were used in conjunction with Phenolyzer (Yang et al. 2015), which is desiged to determine and prioritize which mutations contribute most to the phenotype by comparing the provided HPO numbers to the phenotypes attributed to the gene in which the proband's mutation is located. A VCF file containing the same variants as the BED file used with Phenolyzer was then input into similar programs such as wANNOVAR and PhenIX in order to utilize several sources of analysis (Chang and Wang 2012; Zemojtel et al. 2014). These same VCF files were input into the Omicia Opal system, with similar results (see Supplemental File 4; Rope et al. 2011; Hu et al. 2013; Kennedy et al. 2014).

### Confirmation of Variants

Once a possible disease-contributory mutation was identified, its location was then input into Golden Helix GenomeBrowse, which displayed read information from the BAM files corresponding to each family. All variants of interest were also researched and ruled out as major contributing mutations because of no association with a relevant phenotype. The genic locations of each variant were identified using GEMINI (Paila et al. 2013). Initially, the known functions, phenotypes, and diseases associated with each gene would be researched using the GeneCards online database (Rebhan et al. 1998; Stelzer et al. 2011; Dierking and Schmidtke 2014), which contains information compiled from more than 100 sources. These results were then confirmed by researching the gene in other databases, such as National Center for Biotechnology Information (NCBI), PubMed, and OMIM (Hamosh et al. 2005; Brown et al. 2015). These findings were also compared with the output of the phenotype analysis software. No additional contributing mutations were identified in this individual. The GEMINI query selected no autosomal recessive mutations of interest and 16 rare de novo mutations as mutations of interest. The Phenolyzer, wANNOVAR, and PhenIX outputs all identified a heterozygous missense mutation in *ANKRD11* as the most likely contributing mutation. Sanger sequencing confirmed this variant. The primers used in the procedure are:

 ANKRD11-F-10810 GACTTGTCCTTGAAGCCACTCT
 ANKRD11-R-10810 GGACATGAAGAGCGACTCTGT

## ADDITIONAL INFORMATION

### Data Deposition and Access

The ClinVar (http://www.ncbi.nlm.nih.gov/clinvar/) accession number for the variant is SCV000292225.1. Sequencing data were deposited to the Sequence Read Archive (SRA; http://www.ncbi.nlm.nih.gov/sra/) with the accession number SRR3773726, and the BioSample identifier is SAMN05366061.

### Ethics Statement

Research was carried out in compliance with the Federal Policy for the Protection of Human Subjects 45C.F.R.46. The family was recruited to this study at the Utah Foundation for Biomedical Research (UFBR) where extensive clinical evaluation was performed. Written consent was obtained for phenotyping, use of facial photography, and whole-exome sequencing through Protocol #100 at the UFBR was approved by the Independent Investigational Review Board, Inc.

### Acknowledgments

G.J.L. is supported by funds from the Stanley Institute for Cognitive Genomics at Cold Spring Harbor Laboratory. K.W. is supported by National Institutes of Health (NIH) grant HG006465. We thank the Exome Aggregation Consortium and the groups that provided exome variant data for comparison. A full list of contributing groups can be found at http://exac.broadinstitute.org/about. G.J.L. would like to thank Omicia for access to the Omicia Opal system. The authors acknowledge Jason O'Rawe for bioinformatics support and comments on the manuscript. Justine Coppinger is acknowledged as well for her help with the molecular diagnosis of the child using Affiliated Genetics’ protocols and pipeline for a family study. Finally, we acknowledge clinical input from Dr. Fran Filloux from the University of Utah.

### Conflict of Interest

G.J.L serves on advisory boards for GenePeeks, Inc. and Omicia, Inc. and is a consultant to Good Start Genetics. K.W. is board member and shareholder of Tute Genomics, Inc. R.R. is employee, chief executive officer, and shareholder of Tute Genomics, Inc.

### KBG Syndrome Foundations

Please see: http://www.kbgfoundation.com for the KBG Foundation's Web page and https://www.facebook.com/KGBFdn/ for the KBG Foundation's Facebook page.

### Competing Interest Statement

The authors have declared no competing interest.

## Supplementary Material

Supplemental Material
