# Supplementary material for: KBG syndrome involving a single-nucleotide duplication in ANKRD11
Source: Cold Spring Harb Mol Case Stud. 2016 Nov;2(6):a001131. doi: 10.1101/mcs.a001131 (PMC5111005; doi:10.1101/mcs.a001131)
Supplement: Supplemental Material [file supp_2_6_a001131__index.html]

KBG syndrome involving a single-nucleotide duplication in ANKRD11 — Supplemental Material 

# KBG syndrome involving a single-nucleotide duplication in *ANKRD11*

## Supplemental Material

**Files in this Data Supplement:**

- Supp File 1.mp4
- Supp File 2.zip
- Supp File 3.zip
- Supp File 4.zip
- Supp File 5.zip
- Supp File 6.svg
- Supp Legends.docx
