# Supplementary material for: KBG syndrome involving a single-nucleotide duplication in ANKRD11
Source: Cold Spring Harb Mol Case Stud. 2016 Nov;2(6):a001131. doi: 10.1101/mcs.a001131 (PMC5111005; doi:10.1101/mcs.a001131)
Supplement: Supplemental Material [file supp_mcs.a001131_Supp_File_3_Scripts_Programs.zip › Scripts_Programs/Python/File Descriptions.docx]

File Descriptions:

**(All programs are thoroughly annotated within).**

iPython_Notebook.html – HTML version of the iPython Notebook I used to create the program. I would suggest finding out if we can display this as a webpage, because it looks very elegant and well organized. Can be viewed by clicking on the file name in DropBox, or downloading it, and opening it in Safari on Mac.

iPython_Notebook.pdf – PDF version of the notebook. It is in black and white, and doesn’t look as nice, although it is well organized.

Python Program To Find De Novo and AutRec.py – Program itself.

Files in ‘Inputs’ Folder – Files input into the Python program.

Files in ‘Outputs’ Folder – Files output from the Python program.
