# Supplementary material for: KBG syndrome involving a single-nucleotide duplication in ANKRD11
Source: Cold Spring Harb Mol Case Stud. 2016 Nov;2(6):a001131. doi: 10.1101/mcs.a001131 (PMC5111005; doi:10.1101/mcs.a001131)
Supplement: Supplemental Material [file supp_mcs.a001131_Supp_File_3_Scripts_Programs.zip › Scripts_Programs/Python/iPython_Notebook.pdf]

Program To Find De Novo and Autosomal Recessive Variants

In [45]:
```
#Importing Libraries
import pandas as pd
import numpy as np
import tarfile
import os
import pkg_resources
import sys
```

In [46]:
```
#Displaying Versions:
print("The Python version is %s.%s.%s" % sys.version_info[:3])
print 'The Pandas Version Is: %s' %pkg_resources.get_distribution("pan
das").version
print 'The Numpy Version Is: %s' %pkg_resources.get_distribution("nump
y").version
```

```
The Python version is 2.7.11
The Pandas Version Is: 0.16.2
The Numpy Version Is: 1.9.2
```

In [22]:
```
#CREATES DATAFRAME FROM AVINPUT FILES
proband=pd.read_csv('$PATH/proband.avinput',header=None, delimiter=r"\
t")
mother=pd.read_csv('$PATH/mother.avinput',header=None, delimiter=r"\t"
)
father=pd.read_csv('$PATH/father.avinput',header=None, delimiter=r"\t"
)
sister1= pd.read_csv('$PATH/sister1.avinput',header=None, delimiter=r"
\t")
brother=pd.read_csv('$PATH/brother.avinput',header=None, delimiter=r"\
t")
sister2=pd.read_csv('$PATH/sister2.avinput',header=None, delimiter=r"\
t")
```

FINDING AUTOSOMAL RECESSIVE VARIANTS

In [30]:
```
#SELECT HOMOZYGOUS VARIANTS IN PROBAND
proband_hom = proband.loc[~proband[5].isin(['het'])]
#Select only first four columns.
proband_hom=proband_hom.ix[0:,0:4]

#SELECT HETEROZYGOUS VARIANTS FROM PARENTS

mother_het=mother.loc[~mother[5].isin(['hom'])]
mother_het=mother_het.ix[0:,0:4]

father_het=father.loc[~father[5].isin(['hom'])]
father_het=father_het.ix[0:,0:4]

#FIND VARIANTS THAT ARE HETEROZYGOUS IN BOTH PARENTS
##Inner Merge
parents_het=combine=pd.DataFrame(pd.merge(father_het, mother_het, how=
'inner'))
##Drop Duplicates
parents_het=parents_het.drop_duplicates()


#COMBINE HETEROZYGOUS VARIANTS FROM PARENTS AND HOMOZYGOUS VARIANTS FR
OM CHILD
##Inner Merge
autrec_proto=pd.DataFrame(pd.merge(parents_het, proband_hom, how='inne
r'))


#SUBTRACT SHARED VARIANTS FROM CHILDREN
##Select Homozygous Variants
sister1_hom=sister1.loc[~sister1[5].isin(['het'])]
##Select only first four columns.
sister1_hom=sister1_hom.ix[0:,0:4]
##Do a set subtraction to find homozygous variants in the proband, but
 not the sibling.
autrec_proto=autrec_proto.loc[~autrec_proto[1].isin(sister1_hom[1])]

#REPEATED FOR EACH SIBLING

brother_hom=brother.loc[~brother[5].isin(['het'])]
brother_hom=brother_hom.ix[0:,0:4]
autrec_proto=autrec_proto.loc[~autrec_proto[1].isin(brother_hom[1])]

sister2_hom = sister2.loc[~sister2[5].isin(['het'])]
sister2_hom=sister2_hom.ix[0:,0:4]
autrec_proto=autrec_proto.loc[~autrec_proto[1].isin(sister2_hom[1])]

#Converting to final annovar file and final csv file--Not used for ana
lysis downstream

autrec_final=proband.loc[proband[1].isin(autrec_proto[1])]
autrec_final.to_csv('$PATH/autrec.avinput', index=False, sep='\t')
```

FINDING DE NOVO VARIANTS

In [24]:
```
#Select only first 6 columns of AVINPUT file.
proband2=proband.ix[0:,0:5]
mother2=mother.ix[0:,0:5]
father2=father.ix[0:,0:5]
sister1_2=sister1.ix[0:,0:5]
brother2=brother.ix[0:,0:5]
sister2_2=sister2.ix[0:,0:5]
```

In [25]:
```
#CREATE SET FROM DATAFRAME CONTAINING FIRST SIX COLUMNS OF AVINPUT FIL
E FOR MOTHER AND PROBAND
proband3 = set([ tuple(line) for line in proband2.values.tolist()])
mother3 = set([ tuple(line) for line in mother2.values.tolist()])
#CREATE A DATAFRAME WITH de novo VARIANTS BY SUBTRACTING THE SET WITH
MOTHER'S VARIANTS FROM SET WITH PROBAND'S
de_novo=pd.DataFrame(list(proband3.difference(mother3)))

#REPEATED FOR EACH ADDITIONAL FAMILY MEMBER
de_novo = set([ tuple(line) for line in de_novo.values.tolist()])
father3 = set([ tuple(line) for line in father2.values.tolist()])
de_novo=pd.DataFrame(list(de_novo.difference(father3)))

de_novo = set([ tuple(line) for line in de_novo.values.tolist()])
sister1_3 = set([ tuple(line) for line in sister1_2.values.tolist()])
de_novo=pd.DataFrame(list(de_novo.difference(sister1_3)))

de_novo = set([ tuple(line) for line in de_novo.values.tolist()])
brother3 = set([ tuple(line) for line in brother2.values.tolist()])
de_novo=pd.DataFrame(list(de_novo.difference(brother3)))

de_novo = set([ tuple(line) for line in de_novo.values.tolist()])
sister2_3 = set([ tuple(line) for line in sister2_2.values.tolist()])
de_novo=pd.DataFrame(list(de_novo.difference(sister2_3)))

#SORTING VARIANTS BY CHROMOSOMAL NUMBER AND POSITION
de_novo=de_novo.sort_index(by=[0, 1], ascending=[True, False])

#ENSURES THAT NO VARIANTS IN THE AUTOSOMAL RECESSIVE DATASET ARE IN TH
E DE NOVO DATASET--ADDITIONAL QUAL. CONTROL
de_novo=de_novo.loc[~de_novo[1].isin(autrec_proto[1])]

#SELECTING VARIANTS THAT ARE DE NOVO, BUT ALSO IN THE PROBANDS AVINPUT
 FILE--ALSO QC
de_novo_final=proband.loc[proband[1].isin(de_novo[1])]

#EXPORTING TO AVINPUT
de_novo_final.to_csv('$PATH/de_novo.avinput', index=False, header=Fals
e, sep='\t')
```

EXPORTING BED FILES

In [26]:
```
#Creating a BED file containing regions for de novo variants--tab deli
minated.
de_novo_bed=de_novo_final.ix[0:,0:2]
de_novo_bed.to_csv('$PATH/de_novo.bed', index=False, header=False, sep
='\t')
```

In [27]:
```
#Creating a BED file containing regions for autosomal recessive varian
ts
autrec_final_bed=autrec_final.ix[0:,0:2]
autrec_final_bed.to_csv('$PATH/autrec.bed', index=False, header=False,
 sep='\t')
```
