# Supplementary material for: KBG syndrome involving a single-nucleotide duplication in ANKRD11
Source: Cold Spring Harb Mol Case Stud. 2016 Nov;2(6):a001131. doi: 10.1101/mcs.a001131 (PMC5111005; doi:10.1101/mcs.a001131)
Supplement: Supplemental Material [file supp_mcs.a001131_Supp_File_5_Gemini_Analysis.zip › Gemini_Analysis/Gemini_Upload_Files/autrec_annotated.vcf_summary.html]

VEP summary


Links

- Top of page
- VEP run statistics
- General statistics
- Variant classes
- Consequences (most severe)
- Consequences (all)
- Coding consequences
- SIFT summary
- PolyPhen summary
- Variants by chromosome
- Position in protein

### VEP run statistics

|  |  |
| --- | --- |
| VEP version (API) | 74 (74) |
| Cache/Database | /sonas-hs/lyon/hpc/home/rkleyner/.vep/homo\_sapiens/74 |
| Species | homo\_sapiens |
| Command line options | ``` -i autrec_normalized.vcf --cache --sift b --polyphen b --symbol --numbers --biotype --total_length -o autrec_annotated.vcf --vcf --fields Consequence,Codons,Amino_acids,Gene,SYMBOL,Feature,EXON,PolyPhen,SIFT,Protein_position,BIOTYPE ``` |
| Start time | 2016-02-20 00:33:09 |
| End time | 2016-02-20 00:33:29 |
| Run time | 20 seconds |
| Input file (format) | autrec\_normalized.vcf (VCF) |
| Output file | autrec\_annotated.vcf [text] |

### General statistics

|  |  |
| --- | --- |
| Lines of input read | 450 |
| Variants processed | 308 |
| Variants remaining after filtering | 308 |
| Lines of output written | 308 |
| Novel / known variants | - |
| Overlapped genes | 334 |
| Overlapped transcripts | 1669 |
| Overlapped regulatory features | - |

---

### Variant classes

---

### Consequences (most severe)

---

### Consequences (all)

---

### Coding consequences

---

### SIFT summary

---

### PolyPhen summary

---

### Variants by chromosome

---

### Distribution of variants on chromosome 1

---

### Distribution of variants on chromosome 2

---

### Distribution of variants on chromosome 3

---

### Distribution of variants on chromosome 4

---

### Distribution of variants on chromosome 5

---

### Distribution of variants on chromosome 6

---

### Distribution of variants on chromosome 7

---

### Distribution of variants on chromosome 8

---

### Distribution of variants on chromosome 9

---

### Distribution of variants on chromosome 10

---

### Distribution of variants on chromosome 11

---

### Distribution of variants on chromosome 12

---

### Distribution of variants on chromosome 13

---

### Distribution of variants on chromosome 14

---

### Distribution of variants on chromosome 15

---

### Distribution of variants on chromosome 16

---

### Distribution of variants on chromosome 17

---

### Distribution of variants on chromosome 18

---

### Distribution of variants on chromosome 19

---

### Distribution of variants on chromosome 20

---

### Distribution of variants on chromosome 21

---

### Distribution of variants on chromosome 22

---

### Position in protein
