# Supplementary material for: KBG syndrome involving a single-nucleotide duplication in ANKRD11
Source: Cold Spring Harb Mol Case Stud. 2016 Nov;2(6):a001131. doi: 10.1101/mcs.a001131 (PMC5111005; doi:10.1101/mcs.a001131)
Supplement: Supplemental Material [file supp_mcs.a001131_Supp_Legends_for_KBG.docx]

**SUPPLEMENTARY FILES**

**File 1: Videos in a zip file**

**VIDEO DESCRIPTION**

Parents discuss the proband’s abnormalities with Dr. Gholson Lyon. Included is an additional interview from August 2015, after the parents received the proband’s diagnosis of KBG syndrome.

0:00-0:13 – The proband had an abnormally large fontanelle, which resolved without treatment.

0:13-0:27 – The proband does not appear to have a sacral dimple.

0:27-0:35 – Other than the presence of flat arches, there are no obvious signs of foot abnormalities.

0:35-1:05 – The proband does not look like his other siblings, although there was a resemblance between him and his sister when they were the same age.

1:05-1:12 - Features of proband’s face can be seen, including bushy eyebrows, broad nasal tip, short philtrum, full lips and cupid bow of upper lip.

1:12-1:40 – Video of proband’s behavior. Proband is non-verbal, and hyperactive. He repetitively spins his toy. While playing, he gets up from his chair, walks a few steps, stomps his feet, and sits back down.

1:40 – 2:55 Additional interview in August 2015, after the parents received the proband’s diagnosis of KBG syndrome. The proband develops new mannerisms every four to six months, the most recent being short, hard breaths through the nose and head turning. The proband has had a substantial decrease in the number of seizures after starting an Epidiolex (cannabidiol) treatment (70-80% decrease as described by the parents). The frequency of seizures increased after the proband fell and fractured his jaw.

2:55—4:14The mother describes the proband’s macrodontia. Although the mother and several siblings have large teeth, the macrodontia in the proband does not appear in any other member of the family.

4:14—4:45 The proband’s features are compared to other characteristics usually found in other KBG patients. Unlike most KBG patients, the proband has full lips. Like most KBG patients, the proband has curved pinkies (diagnosed as clinodactyly), which are often found in KBG patients.

4:45—5:11 Although the proband has relatively short toes, this trait may have been inherited from the father. The proband also has curved toenails, which commonly appear in autistic children.

File 2. VCF FILES in a zip-compressed folder

proband.vcf

Unaffected_brother.vcf

Unaffected_father.vcf

Unaffected_mother.vcf

Unaffected_sister1.vcf

Unaffected_sister2.vcf

File 3. Program Scripts and Outputs used in the analysis, uploaded as a zip file, containing the files below.

**Python Folder:**

iPython_Notebook.html – HTML version of the annotated iPython Notebook used to create the program, which can be viewed by opening the file in a web browser such as Safari or Firefox.

iPython_Notebook.pdf – PDF version of the annotated iPython Notebook.

Python Program To Find De Novo and AutRec.py – Program used to identify *de novo* and autosomal recessive variants.

Files in ‘Inputs’ Folder – Files input into the Python program.

Files in ‘Outputs’ Folder – Files output from the Python program.

**Variant Calling Folder:**

Sample_Pipeline.sh – Annotated script used to analyze raw read (fastq) files to VCF files.

Final_VCF Prep.sh – Script used to create a single VCF file from the analysis pipeline’s outputs.

File 4. Excel SPREADSHEETS ILLUSTRATING VAAST RESULTS AMONG THE VARIOUS QUADS in a zip folder, run using the Omicia Opal software.

Supplementary Table 1: Omicia ‘QUAD’ analysis run with Sister 1 designated as the unaffected sibling. **Supplementary Table 1.xlsx**

Supplementary Table 2: Omicia ‘QUAD’ analysis run with Sister 2 designated as the unaffected sibling. **Supplementary Table 2.xlsx**

Supplementary Table 3: Omicia ‘QUAD’ analysis run with the brother designated as the unaffected sibling. **Supplementary Table 3.xlsx**

File 5. GEMINI_Analysis.zip

de_novo_interest.bed – BED file including variants which were selected using the GEMINI query discussed in the paper (*gemini query -q "select chrom, start, end from variants where qual>=120 AND (cadd_scaled>20 OR cadd_scaled is NULL) AND in_exac=0 order by chrom, start" denovo.db)*.

de_novo_interest.vcf – BED file including variants which were selected using the GEMINI query discussed in the paper (*gemini query -q "select chrom, start, end from variants where qual>=120 AND (cadd_scaled>20 OR cadd_scaled is NULL) AND in_exac=0 order by chrom, start" denovo.db)*.

**Denovo_Autrec_VCF**

autrec.vcf – VCF including all autosomal recessive variants.

Denovo.vcf – VCF including all *de novo* variants.

**Gemini_Upload_Files -** Includes a script used to ‘upload’ files to GEMINI, through which a database of variants. Also includes the files created when variant databases were created using GEMINI.

**Variant Database:**

autrec.db – GEMINI database of autosomal recessive variants.

denovo.db – GEMINI database of *de novo* variants.
